# Downward accountability to beneficiaries in social enterprises: do partnerships with nonprofits boost it without undermining accountability to other stakeholders?

**DOI:** 10.1007/s11846-021-00485-6

**Published:** 2021-08-16

**Authors:** Maria José Sanzo-Pérez, Marta Rey-García, Luis Ignacio Álvarez-González

**Affiliations:** 1 Department of Business Administration, University of Oviedo, Oviedo, Spain; 2 Department of Business Administration, University of A Coruña, A Coruña, Spain

**Keywords:** Social enterprises, Partnerships, Stakeholder management, Accountability, L31, M13, O35

## Abstract

The hybrid nature of social enterprises gives them a high potential for developing social innovations, but at the same time leads to tensions within these organizations. The barriers they face to gain access to traditional sources of funding are pushing social enterprises to reinforce their business models and rely more on commercial activities, and this fact increases the risk of mission drift and can weaken accountability towards beneficiaries of the social mission in favor of dominant stakeholders such as funders or clients of the commercial activities. Our research attempts to analyze whether partnerships between social enterprises and nonprofits strengthen accountability to beneficiaries without hindering accountability to other stakeholders, thus allowing both social and economic objectives to operate together. Based on a survey with a sample of social enterprises partnering with nonprofits, results reveal that as the partnership moves along a collaboration continuum to a transformational stage, accountability to beneficiaries is encouraged, whereas accountability to other types of stakeholders is also improved or, at least, not affected.

## Introduction

Social enterprises (SEs), conceived as “organizations whose purpose is to achieve a social mission through the use of market mechanisms” (Ebrahim et al. 2014: 82), are attracting growing research interest (Berbegal-Mirabent et al. 2021; Mongelli et al. 2019; Muñoz and Kimmitt 2019; Tykkyläinen and Ritala 2021). The term SE can refer to different types of organizations, but the following criteria are usually employed to characterize them (European Commission 2015): (1) they are engaged in an economic activity, (2) they pursue an explicit and primary social aim, (3) they have limits on distribution of profits and assets, and (4) they are independent and participatory in terms of governance.

The current focus of scholars and practitioners on SEs has been triggered by the fact that the dual nature of SEs gives them a high potential to foster the so called social innovation (Bouchard 2012), a topic that has gained momentum during the last years, as (1) industrial economies translate to knowledge and service-based societies, leading to the opening of the innovation process to society (Grimm et al. 2013), and (2) traditional welfare systems reveal insufficient to address the serious social and environmental challenges around the world (Anheier et al. 2019). The crisis caused by the COVID-19 pandemic has accelerated both trends, showing the vulnerability of many apparently solid systems, and bringing to light numerous initiatives based on a community model that combines the local dimension with the globalization of knowledge and interconnectivity (Dahlek et al. 2021).

But SEs, which mostly consist of SMEs (Shaw and Bruin 2013), face important barriers to grow and scale-up their activities. On the one hand, reliance on the public sector has proved unsustainable due to austerity measures. On the other hand, they face restrictions on profit distribution, which makes access to traditional sources of funding, particularly equity, problematic. In this regard, McDermott et al. (2018: 127) recognize “three challenges that social entrepreneurs face while mobilizing resources; the first factor relates to the difficulties faced while attempting to attract and retain talent, for the financial reasons mentioned above. Second, there are fewer financial institutions willing to work with organizations without a clear profit motive. Third, access to startup capital is not as readily available for mission focused social enterprises when compared with commercially oriented ventures.”

These barriers are pushing SEs to rely more on market income. As Ramus and Vaccaro (2017: 309) posit, “social enterprises have been overexposed to legislative, cultural, and market pressures to increase commercialization and efficiency. Consequently, a large number of these ventures have shifted away from their original social mission in search of increased revenues.” In this context, the risk of mission drift increases since they can be prone to prioritizing financial sustainability (and therefore accountability to providers of financial resources and revenues) over their social goals (and accountability to beneficiaries could be damaged, especially if they differ from customers).

Scholars have extensively highlighted that the combination of business and social logics involves the appearance of tensions (Battilana and Lee 2014; Ebrahim et al. 2014; Siegner et al. 2018; Smith et al. 2013). Yet, a more recent research stream has changed its focus to a more positive side of hybridity centered on the “the potential inherent in SEs for fostering inclusion, triggering positive societal transformation and generating impact by virtue of their commercial activities and exposure to market pressures” (Mongelli et al. 2019: 302). Regarding this approach, Battilana and Lee (2014: 424) had already noted that “[p]ast research has focused largely on tensions that threaten the sustainability of hybrids […] Less studied, and warranting greater attention, are the generative possibilities of social enterprise, and hybrid organizations in general. Scholars have proposed that the combination of disparate organizational elements may, under certain conditions, provide greater opportunities for discretion and change […]. We suggest that the realization of these outcomes is contingent upon organizational factors—organizational activities, workforce composition, organization design, inter-organizational relationships, and organizational culture—that shape how organizations experience both the conflictual and generative aspects of the combination of forms.”

Our research adopts this perspective and responds to the recent calls made for analyzing the micro-foundations of the organizational strategies and practices undertaken to balance social and economic objectives (Muñoz and Kimmitt 2019). Past studies have underlined the role of stakeholder engagement and, specifically, partnerships between SEs and nonprofits as a means to achieve this goal (Ramus and Vaccaro 2017; Vickers et al. 2017), but we attempt to go one step further and analyze the characteristics that the partnership should present to improve accountability to beneficiaries without hindering accountability to other stakeholders. Following Battilana and Lee (2014: 420–421), “[c]lose relationships with organizations embedded in more established sectors will likely influence social enterprises’ ability to achieve their social mission [..], but the consequences of these relationships for social enterprises are not well understood. [..] By engaging in close partnerships with typical for-profits or not-profits, do social enterprises risk compromising their hybrid nature? Future research will need to study social enterprises’ inter-organizational networks and their evolution over time in order to help address these questions.”

Particularly, as partnerships are extremely diverse in their objectives, characteristics, value generated, and degree of embeddedness in the partners’ missions and core activities, we will characterize the partnership according to its position along a collaboration continuum defined in terms of the strategic nature of the collaboration, degree of relational development, change involved, and innovation and type of value generated, as well as the type of resources provided by each of the partners (Austin and Seitanidi 2012a). Then, we will examine the influence of this position on the accountability that SEs maintain towards different types of stakeholders, including donors, employees, public administrations, customers, and beneficiaries.

Moreover, we will also control for the effects associated with the type of SE considered. Hybridity may be translated into different models, depending on factors such as whether customers and beneficiaries are the same target or not, or whether the enterprise attempts to create social value through the same activities carried out for obtaining financial outcomes or, on the contrary, creating social value requires different activities (Santos et al. 2015). Specifically, we will differentiate between (1) work integration SEs (WISEs), organizations in which customers and beneficiaries are different groups and that carry out different activities to serve each of these targets, and (2) a typology of SEs that depend more on market income, providing environmentally friendly products and services, renewable energy or other innovative offerings at a market price, and that seek to better integrate their social mission with their entrepreneurial activity, thus adopting other hybrid forms different from WISEs.

The contribution of this research is twofold. Firstly, it contributes to SE and hybrid organization literature by extending the range of different configurations of strategic conditions previously identified to balance social and economic logics (Muñoz and Kimmitt 2019), highlighting the role that partnerships between SEs and nonprofits (NPOs) can play in achieving this objective. Secondly, it also contributes to legitimacy literature by exploring a means through which multi-stakeholder organizations can gain legitimacy, because “we know very little about legitimacy acquisition by SoEs [social enterprises] and, more importantly, their situated managerial organizing practices that could potentially help them to acquire legitimacy” (Sarpong and Davies 2014: 23).

The presentation of the research is structured in the following manner. We first provide the conceptual framework and justify our basic hypotheses. Next, we detail the methodology, present the empirical results, and discuss their implications. Finally, limitations and further research directions are also included.

## Theoretical framework

### Social enterprises as hybrid organizations

There is not a unique concept for SEs or social entrepreneurship (Saebi et al. 2019), in such a way that the term ‘social enterprise’ has been employed “within academia as an “umbrella” construct, with wide scope and ambiguous boundaries” (Battilana and Lee 2014: 406). Despite this ambiguity, a SE is usually defined as a hybrid organization. Hybrid organizations (e.g., mission-driven businesses, social enterprises, public–private partnerships) are characterized by a combination of public and private organizing logics (Jay 2013), and SEs, conceptualized as “organizations whose purpose is to achieve a social mission through the use of market mechanisms” (Ebrahim et al. 2014, p. 82), involve organizations that manage two apparently opposite goals, social and economic value creation.

From an institutional perspective, the European Commission (2012) states that SEs “combine societal goals with an entrepreneurial spirit” (para. 1) and defines a SE as “an operator in the social economy whose main objective is to have a social impact rather than make a profit for their owners or shareholders. It operates by providing goods and services for the market in an entrepreneurial and innovative fashion and uses its profits primarily to achieve social objectives. It is managed in an open and responsible manner and, in particular, involves employees, consumers and stakeholders affected by its commercial activities” (para. 3).

The European Commission (2012) recognizes that there is no single legal form for SEs, but that most of them operate in the following four fields: (1) Work integration (training and integration of unemployed and/or disabled people), (2) personal social services (health, education, childcare services, services for elderly people, or aid for disadvantaged people), (3) local development of disadvantaged areas (SEs in remote rural areas, neighborhood development/rehabilitation schemes in urban areas, development aid and development cooperation with third countries), and (4) other including recycling, environmental protection, sports, arts, culture or historical preservation, science, research and innovation, consumer protection and amateur sports.

From the scholars’ perspective, the recent systematic literature review undertaken by Saebi et al. (2019: 72) remarks that “the dual mission of social and economic value creation reflects the core characteristic of SE” and that is “the attempt to combine social and economic missions that makes SE unique and sets it apart from activities dominated by primarily an economic mission (e.g., commercial entrepreneurship, CSR [corporate social responsibility]) or social mission (e.g., nonprofit/philanthropic organizations)” (p. 73).

Adopting an organizational level of analysis, scholars have also stressed that SEs have the potential to create significant opportunities for innovation and value creation, but also suffer from the appearance of tensions and conflicts (Battilana and Lee 2014; Ebrahim et al. 20l4; Muñoz and Kimmitt 2019; Ramus and Vaccaro 2017; Siegner et al. 2018). These divergences can appear internally, in managing organizational identity, resource allocation, and decision-making, and also externally, in managing relationships with different kinds of environments, resulting in legitimacy problems and difficulty in the acquisition of resources. The potential consequence of these conflicts is the existence of goal displacement towards the better-established o dominant form (Battilana and Lee 2014), usually the business model (Ramus and Vaccaro 2017).

To face these tensions, inter-organizational relationships (Battilana and Lee 2014: 420–421), “relationships with different types of external stakeholders, partnerships, and outsourcing relationships, and governance systems design”, “structures and processes that are designed to ensure accountability, transparency, responsiveness, rule of law, stability, equity and inclusiveness, empowerment, and broad-based participation” (IBE-UNESCO, (2012) para. 1) play a critical role in hybrid organizing in SEs, along with the design of the formal organizational structure, and incentive/control systems. In this respect, “social enterprises that combine business and charity at their core face unique governance challenges related to joint accountability to both social and economic objectives […] thereby resisting pressures to “drift” toward either social or economic objectives at the expense of the other” (Battilana and Lee 2014: 419). Next section will address this topic.

### Accountability in social enterprises

Accountability has been defined as “the means through which individuals and organizations are held externally to account for their actions and as the means by which they take internal responsibility for continuously shaping and scrutinizing organizational mission, goals, and performance” (Ebrahim 2003: 194). Hybridity makes accountability of SEs particularly challenging, since these organizations must articulate different accountabilities, most notably upward accountability (to investors/donors) and downward accountability (to beneficiaries, clients and other non-shareholders) (Ebrahim et al. 2014).

Hybridity may put downward accountability towards beneficiaries at risk. Beneficiaries show a very weak power position, due to the asymmetry of their relationships with the organization: they are in a ‘take it or leave it’ relationship (Ebrahim 2003). And, as Battilana and Lee (2014: 414–415) state, “organizations more readily comply with the demands stemming from external constituencies on which they depend for key resources, while they are more likely to resist the demands from constituencies on which they do not depend.” As funders and customers largely influence accountability as dominant stakeholders (Bradford et al. 2018), we argue that reinforcing the inherently weak accountability of SEs to beneficiaries is pivotal to really trigger societal transformation, especially in those SEs in which customers and beneficiaries are different targets and it is therefore more likely that the organization favors “the interests of customers over the interests of the beneficiaries on which the organization does not depend financially” (Battilana and Lee 2014: 415).

Goal displacement may hinder the legitimacy of the SE. Legitimacy, understood as “a generalized perception or assumption that the actions of an entity are socially desirable, proper or appropriate within some socially constructed system of norms, value, beliefs and definitions” (Suchman 1995: 574), is essential in SEs to obtain and mobilize the resources required for operating (Sarpong and Davies 2014). And, as current increased competition for resources may favor accountability to funders and customers in detrimental of accountability to beneficiaries, the relevance of identifying potential strategies that help SEs acquire and reinforce their legitimacy, especially in the eyes of the less powerful stakeholders, will become greater.

Among the different means to gain legitimacy, previous research works have insistently noted that partnerships (i.e. formal and informal relationships, networks, and alliances that SEs build and develop with businesses, public administrations, and civil society groups) can enhance not only their access to economic resources but also their credibility and legitimacy (Sarpong and Davies 2014). The key therefore is to identify what organizational characteristics should present these collaborations to really help SEs in their processes of legitimacy building.

### Partnerships between social enterprises and nonprofits

Partnering with NPOs could be an alternative to counteract the dominance of a business logic in SEs, by strengthening the role of those stakeholders linked to the social mission (Sarpong and Davies 2014; Ramus and Vaccaro 2017) Although SEs share some characteristics with NPOs, they are different from nonprofit organizations, as they also include a commercial activity at their core (not as a peripheral activity). So, the collaboration can be considered as a special type of cross-sector partnership in which the SE adopts the role of the organization that develops the business activity.

As occurs with the SE concept, the label ‘partnership’ is also an umbrella construct that encompasses very different alternatives in terms of value generated, risks involved, and governance structures. Furthermore, it is not unusual the use of this term as a synonym for collaboration in a broad sense. In our research, we assume that cross-sector partnerships “mean relatively intensive, long-term interactions between organizations from at least two sectors (business, government, and/or civil society) aimed at addressing a social or environmental problem” (Clarke and Crane 2018: 303). Overall, cross-sector partnerships, and particularly partnerships between businesses and NPOs, have been encouraged due to the different types of value that can be derived from them (Austin and Seitanidi 2012b): associational value (visibility, credibility), transferred value (cash, in-kind gifts, volunteer capital, complementary and organization-specific assets, …), interaction value (learning, access to networks), and synergistic value (innovation, shared leadership).

In the case of SEs, we expect that partnering with nonprofits could serve to countervail the risk of mission drift in the SE, since NPOs (as representative organizations of beneficiaries) can reinforce the role of beneficiaries, strengthen legitimacy of the SE, and help the SE identify, contact, and build stronger relationships with these targets. For example, and supporting this expectation, Ramus and Vaccaro (2017) have compared the strategies followed by two Italian WISEs that had experienced a mission drift, finding better results in the SE that elected to address this problem through a process of multi-stakeholder engagement, particularly focused on collaborating with actors from the nonprofit sector. Their results show that “internal strategies based on social accounting alone do not support SEs to counterbalance mission drift, once occurred” and that “the main mechanism to address mission drift is stakeholder engagement” (Ramus and Vaccaro 2017: 319).

But not all partnerships are equal, and their impact on reinforcing the role of beneficiaries in the SE can vary significantly. In this sense, Le Ber and Branzei (2010: 603) have analyzed the role of beneficiaries in the value creation process in cross-sector partnerships, and posited that “beneficiaries often remain marginalized during value creation processes and thus many of their potential contributions may fail to materialize.” As Sarpong and Davies (2014: 27) also posit, “we observed that these serial collaborations on their own did not lead to the acquisition of legitimacy by the SoEs. Rather, legitimacy could be extracted from these collaborations if the members of the network can be strategically co-opted into making philanthropic and cultural investments in the activities and social missions of the enterprises.”

Thus, as it was above-mentioned, the label ‘partnership’ embraces very different alternatives of collaborations. Although this term is usually used to refer to relatively intensive and long-term interactions, the ambiguity associated with ‘relatively’ still leads to a broad range of possibilities. Scholars have recurrently used a ‘collaboration continuum’ scheme to identify different types of business-NPO partnerships. One of the best-known models is the so-called ‘collaborative value creation’ framework (Austin and Seitanidi 2012a, b), which identifies four basic categories of collaborations, i.e. philanthropic, transactional, integrative, and transformational partnerships. Except for philanthropic collaborations, the remaining types could be fully included within the conceptualization of partnership considered in our research.

‘Philanthropic collaborations’ are characterized by unilateral directionality of the resource flow (basically cash), from the company (the donor) to the nonprofit (the recipient). The degree of interaction between them is generally limited and their activities rather independent. Associational value is generated, but this type of collaboration “does not add any more value than what would come from any other cash donor” (Austin and Seitanidi (2012a: 738).

In the case of ‘transactional collaborations’, Austin and Seitanidi (2012a: 739) note that they “include highly developed employee volunteer programs, CRM [cause-related marketing], event and other sponsorships, name and logo licensing agreements, various certification arrangements, and other specific projects with clear objectives, assigned responsibilities, programmed activities, and predetermined timetables.” The directionality of the resource flow becomes bilateral. There is higher resource complementarity, and the nature of transferred resources involves more specialized assets. The benefits to the organizations tend to be more direct, but it is less clear the realization of improved societal welfare.

In ‘integrative collaborations’ the partners’ missions, values, and strategies are more congruent as a result of developing closer relationships and greater trust. The collaboration is seen as an integral part of the strategy of each organization and generating societal value gains greater importance. Core competencies are increasingly employed, using them not in an isolated way, but in combination. Interaction value emerges as a more significant benefit.

Finally, in ‘transformational collaborations’ the end beneficiaries take a more active role, as the aim is to create disruptive social innovations. These collaborations are characterized by interdependence and collective action. Their effects “would not only be in social, economic, or political systems but also change each organization and its people in profound, structural, and irreversible ways” (Austin and Seitanidi 2012a: 744).

The type of collaboration is defined by its position along the ‘collaboration continuum’, which in turn depends on the following factors (Austin and Seitanidi 2012a): (1) level of engagement (from low to high), (2) importance to mission (from peripheral to central), (3) magnitude of resources (form small to big), (4) type of resources (from money to core competencies), (5) scope of activities (from narrow to broad), (6) interaction level (from infrequent to intensive), (7) trust (from modest to deep), (8) internal change (from minimal to great), (9) managerial complexity (from simple to complex), (10) strategic value (from minor to major), (11) co-creation of value (from sole to conjoined), (12) synergistic value (from occasional to predominant), (13) innovation (from seldom to frequent), and (14) external system change (from rare to common). These factors move from low to high levels as the partnership reaches the integrative and, specially, the transformational stage.

We can expect that the kind of partnership can also affect the relationships that the SE maintains with its stakeholders, and therefore its accountability strategy. The analysis undertaken by Ramus and Vaccaro (2017) have showed that the closeness of the relationship allows the SE to rationalize its priorities, re-conceptualize the understanding of the values and motivations at the core of its mission, operationalize the pro-social goals into organizational practices, acquire the technical and managerial skills from the stakeholders needed to scale the social impact, as well as foster the collaboration of other potential partners. In a similar way, McDermott et al. (2018: 126) posit that “[i]t was not enough to simply get stakeholders “to the table”; there was also a concerted effort to ensure that stakeholders remained engaged over the long-term”, by effective communication mechanisms, using core capabilities of the partner, or encouraging co-creation of solutions for the social issue, among other activities.

Those partnerships positioned in the transformational stage are characterized by the presence of co-creation of value and social innovations (Austin and Seitanidi 2012a), that “involve a higher degree of bottom-up and grass-roots involvement than other types of innovation” (Anheier et al. 2019: 19). As nonprofits usually maintain direct and close paths of communication with beneficiaries, since they are governed by the principal stakeholders, if a SE is engaged in a strategic partnership with a nonprofit, it can be more likely that the nonprofit will be willing to promote the engagement of the beneficiaries with the SE activities and processes, as well as co-creation processes, strengthening the power position of beneficiaries (and also the role of customers in those SEs in which beneficiaries and customers are the same targets), as well as the SE accountability toward them.

For beneficiaries, the NPO may represent a highly credible source, and as noted by McDermott et al. (2018: 129), “[s]takeholders who participate actively in the CSSPs [cross-sector social partnerships] are formally and informally engaged in awareness building narratives through their communication activities with other stakeholders. These communication activities may also be thought of as “free marketing,” where awareness of the CSSP and the social or environmental issue is articulated from a credible source.” We propose the following hypothesis:

H1: As the SE-nonprofit partnership evolves along the collaboration continuum toward the transformational stage, downward accountability to beneficiaries in SEs will be reinforced.

Moreover, we can also expect that as the collaboration moves to the transformational stage, and the projects become more intertwined with the SE’s core actions, the extent to which its social and commercial activities are integrated will also increase, allowing the SE to achieve its social and commercial goals at the same time. The findings provided by Ramus and Vaccaro (2017: 320) suggest that to develop a successful strategy to face a mission drift “stakeholder engagement should be integrated with the overall strategy of a venture rather than being free of any strategic motivation and instrumental intention,” so it is likely that in such a type of transformative partnerships there are not tradeoffs between accountability to beneficiaries and accountability to other categories of stakeholders. Consequently,

H2: As the SE-nonprofit partnership evolves along the collaboration continuum toward the transformational stage, accountability to other types of stakeholders in SEs (funders/donors, employees, clients, and public administrations) will not be damaged.

## Methodology

### Data collection and sample description

We conducted a quantitative study by means of a survey to a sample of SEs in Spain. With the objective of controlling different hybrid models, we collected a convenience database comprised of two types of SEs.

The first one includes the work integration SEs (WISEs). A WISE is defined in Spain as a “legally constituted commercial society or cooperative society that […] performs any economic activity of production of goods and services, whose social purpose is the integration and socio-labor training of people in situations of social exclusion as transit to ordinary employment” (Art. 4 of Law 44/2007, of December 13, to regulate work integration SEs). In Spain WISEs are consistently entitled to public subsidies in the form of tax benefits, particularly through rebates in their social security contributions.

The second type refers to a different typology of SEs that mostly relies on market income, adopt commercial legal forms, and take advantage of digital transformation and other sources of social innovation. Whereas WISEs have a long tradition in Spain, this second category of SEs has flourished since the beginning of the deep economic crisis originated in 2008.

As no institutional public register exists, it was necessary to generate an ad-hoc database for the present study, using multiple secondary information sources; from private, but partial, directories of social enterprises or social innovations, to social entrepreneurship crowdfunding platforms, awards for social innovations or social entrepreneurship transformation projects, or networks/forums of this type of enterprises. Overall, 35 secondary sources were combined, categorized in 7 main groups: (1) directories of national and regional associations of Spanish WISEs (e.g. Federación de Asociaciones Empresariales de Empresas de Inserción) or social economy (e.g. Confederación Empresarial Española de la Economía Social), (2) private directories of SEs (e.g. ESADE Directory); (3) directories of social innovation organizations (e.g. Digital Social Innovation Report), (2) networks/forums of social innovation (e.g. Social Innovation Community), (4) networks of social entrepreneurship projects (e.g. Ashoka), (5) solidarity crowdfunding platforms (e.g. Goteo), (6) awards for innovation, entrepreneurship or social transformation (e.g. European University Awards to young social entrepreneurs) and (7) online platforms of solidarity economy (e.g. www.economiasolidaria.org).

This process resulted in an initial database of 345 SEs. We followed the Tailored Design Method (Dillman et al. 2014), that emphasizes the importance of engendering the respondents’ trust in that the expected benefits of their answers would outweigh the costs of responding. With the aim of stimulating their participation in the survey, we contacted all of them by telephone and provided them with information about the study, including a promise of an executive summary of the main results of the survey. After this encouragement process, we sent an online questionnaire to the person in charge of the daily decisions of each organization that accepted to collaborate (294 SEs). The final sample is comprised of 200 Spanish SEs (sample error of ± 4.5% at a 95% confidence level). Of them, 159 (79.5%) collaborated or had collaborated with a NPO different from those NPOs that could eventually have promoted the creation of the SE. Table 1 includes the description of the sample.

**Table 1:** Sample description

Variables	Description	Sample (N = 200)	Sample (N = 159)
Typology of SE	New social enterprise WISE	52.0% 48.0	51.6 48.4
Legal form	Commercial societies Social economy societies	84.0 16.0	85.5 14.5
Type of promoters	Natural person Association Foundation Public Administration Commercial/trading businesses Social economy enterprise Religious entity	47.0 19.5 23.5 2.0 6.5 12.0 6.0	47.8 21.4 23.3 1.9 5.0 11.9 5.7
Main area of social activities (ICNPO)	Culture/recreation Education Research Health Environment Local development and housing Law, advocacy and politics International development cooperation Religion Business, professional associations, unions	18.0 35.0 15.0 19.5 44.0 34.0 13.0 12.5 1.0 14.5	20.8 39.6 17.6 18.9 44.0 32.7 15.7 14.5 0.6 15.1
Size	Micro-sized Small-sized Medium-sized Large/mega-sized	52.0 37.5 8.5 2.0	51.6 37.1 8.8 2.5

To assess the nonresponse bias, we compared early versus late respondents (Armstrong and Overton 1977). We identified two groups of respondents by level of effort. The first group (early respondents) involved 120 SEs that sent back their response after a unique previous contact. The second group included 80 enterprises from which we obtained the data after an extra effort of nonresponse follow-up. The estimation of a two-sample (independent) t-test reveals no statistically significant differences (*p* > 0.05) between both groups in practically all variables of the model.

### Measuring model variables

To measure the accountability of the SE, we use five dependent variables. Each one measures the self-perceived accountability towards each of the five stakeholders considered in this research: beneficiaries of the social mission (BENEFICIARIES), employees (EMPLOYEES), investors/donors (DONORS), customers of the commercial activity (CUSTOMERS), and public administrations (PUBLICAD). Each of these variables was calculated as a mean of two items (measured in a seven-point Likert scale): (1) to what extent each stakeholder contributes to define what the success of the social enterprise is (ranging from “1 = this stakeholder does not contribute at all” to “7 = this stakeholder contributes in a very significant manner”), and (2) to what extent the SE perceives it is accountable to each stakeholder (from “1 = it is not accountable at all” to “7 = it is largely accountable”). The Appendix shows their respective frequencies.

These measures were developed specifically for this research, as a proxy of accountability considering the theoretical framework, and particularly the definition of accountability proposed by Ebrahim (2003: 194): “the means through which individuals and organizations are held externally to account for their actions and as the means by which they take internal responsibility for continuously shaping and scrutinizing organizational mission, goals, and performance.” We attempted to assess to what extent the respondents perceived that each of the stakeholders plays a relevant role in defining the objectives, priorities, or types of performance of the enterprise, as a measure of the power position of each of these stakeholders and therefore their potential influence, and the degree to which the social enterprise is accountable for their actions to each of the stakeholders.

To measure the effect of the SE-NPO partnership on each dependent variable, we used two constructs as predictor variables (Appendix), i.e. (1) the overall position of the partnership along the collaboration continuum, and (2) the specific type of resources provided by each of the partners. To identify those SEs that had collaborated with an NPO, we asked respondents to indicate whether their companies collaborated (or had collaborated) at some point of the last five years with a nonprofit organization, different from those that could have eventually promoted the creation of the enterprise, and without considering the commercial relationships established in exchange for a monetary payment. A dichotomous variable, in which “1” meant that the enterprise had collaborated with an NPO, and “0” meant that it had not, was used. In those cases in which the SEs had collaborated with several nonprofits, we asked them to select the nonprofit that they considered as the main partner.

Respondents who answered in the affirmative that filter question also assessed (in a seven-point Likert scale –see Appendix) the type of partnership in terms of the fourteen characteristics used by Austin and Seitanidi (2012a) to describe the collaboration continuum framework. We employed their model because it represents one of the most referred frameworks, and it provides a detailed range of items to measure the position of the partnership along this continuum. We decided to use the continuum framework instead of asking respondents to select the type of collaboration they maintain with NPOs (i.e. philanthropic, transactional, integrative, and transformational partnerships) because “it recognizes that collaborations are dynamic and that stages are not discrete points; conceptually and in practice a collaborative relationship is multifaceted, and some characteristics may be closer to one reference stage while other traits are closer to another” (Austin and Seitanidi 2012a: 737).

The descriptors were included in the questionnaire through using directly the items proposed by Austin and Seitanidi (2012a), except for the item ‘type of resources.’ In order to assure that respondents did not link the term ‘resources’ exclusively to cash, we decided to analyze these descriptor in further depth, asking SEs to indicate to what extent each one of the partners had provided five types of resources to the partnership: (1) human resources (employees and/or volunteers), (2) monetary resources (cash), (3) in-kind (either infrastructure such as furniture, accommodation, technical equipment, or office supplies, services, etc.), (4) relational-based resources (networks of contacts, information and knowledge about the environment, brand image or reputation), and (5) internal capabilities (technical training, experience in management, experience in project development, promotion of projects). With regard to the remaining 13 items of the original scale, we also merged the items ‘innovation’ and ‘co-creation’ into one single item.

We carried out a K-means cluster analysis to identify homogeneous groups of collaborations based on the type of resources provided (Table 2). Four clusters emerged from the analysis. The first group (19.5% of the partnerships) is characterized by the lower scores in the five categories of resources. Cluster 2 (23.9%) includes partnerships in which SEs and nonprofits provide the five types of resources to a greater extent (with the exception of cash in the case of SEs). In Cluster 3 (35.2%), nonprofits and SEs stand out, respectively, for their contribution in terms of human resources and relational-based resources. Finally, Cluster 4 (21.4%) is comprised of partnerships in which SEs seem to play the traditional role of provider of monetary resources (although the scores corresponding to relational-based and internal capabilities are also high, they are lower than in Cluster 2), and nonprofits contribute less than in Clusters 2 and 3, with the exception of internal capabilities. Four dichotomous variables (CLUSTER1, CLUSTER2, CLUSTER3 and CLUSTER4) were created. In each of them a value of “1” means that the collaboration belongs to that particular cluster, and “0” indicates it does not. In order to avoid collinearity problems, we have introduced in the regression models the three largest clusters: CLUSTER2, CLUSTER3 and CLUSTER4.

**Table 2:** Types of partnerships in terms of the resources provided

Type of Resources	Cluster1 (19.5%)	Cluster2 (23.9%)	Cluster3 (35.2%)	Cluster4 (21.4%)	Sig.
*Social enterprises*					
Human resources	2.87(1.893)	6.32(0.842)	5.39(1.410)	4.97(1.605)	0.000
Financial resources	2.06(1.389)	4.63(1.567)	2.29(1.275)	5.18(1.466)	0.000
In-kind resources	2.19(1.282)	5.18(1.233)	3.37(1.406)	3.38(1.809)	0.000
Relational-based resources	2.99(1.292)	5.98(0.625)	4.79(1.078)	5.27(1.023)	0.000
Internal capabilities	2.80(1.226)	5.96(0.839)	4.69(1.259)	5.00(0.772)	0.000
*Nonprofits*					
Human resources	2.17(1.577)	6.03(0.854)	4.34(1.517)	3.35(2.028)	0.000
Financial resources	1.60(1.380)	4.55(2.089)	3.52(1.945)	1.29(0.524)	0.000
In-kind resources	2.28(1.478)	5.34(1.197)	3.87(1.625)	2.00(1.255)	0.000
Relational-based resources	3.16(1.327)	5.75(1.137)	5.06(1.289)	4.77(1.392)	0.000
Internal capabilities	2.62(1.308)	5.61(1.029)	4.25(1.345)	4.28(1.479)	0.000

* Each cell includes the mean values (measured in a seven-point Likert scale) and the standard deviations (in brackets)

Finally, due to the high diversity that characterize SEs, we considered as control variables the characteristics described in Table 3.

**Table 3:** Control variables

Variables	Sample (N = 159)	Description	
Typology (TYPE)	New social enterprise (TYPE = 0) WISE (TYPE = 1)	51.6% 48.4	We differentiate between WISEs and ‘new’ SEs created mainly after 2008
Legal Form (LEGFORM)	Commercial societies (LEGFORM = 0) Social economy societies (LEGFORM = 1)	85.5 14.5	Commercial businesses include corporations, limited liability societies, single-member limited societies, other SE forms refer to cooperatives, worker-owned companies, other
Type of Promoters	Natural person (PR_NP) Association (PR_ASSOC) Foundation (PR_FOUND) Public Administration (PR_PA) Commercial/trading businesses (PR_COMM) Social economy enterprise (PR_SE) Religious entity (PR_REL)	47.8 21.4 23.3 1.9 5.0 11.9 5.7	Type of promoters/founders of the SE
Main Area of Social Activities (ICNPO)	Culture/recreation (ACT_CUL) Education (ACT_EDU) Research (ACT_RES) Health (ACT_HEALTH) Environment(ACT_ENV) Local development and housing (ACT_HOUS) Law, advocacy and politics (ACT_ADV) International development cooperation (ACT_COOP) Religion (ACT_REL) Business, professional associations, unions (ACT_ASSO)	20.8 39.6 17.6 18.9 44.0 32.7 15.7 14.5 0.6 15.1	We characterized enterprises according to the area of activity complementary to their social core, based on the International Classification of Nonprofit Organizations (ICNPO)
Size	Micro-sized (MICRO) Small-sized (SMALL) Medium-sized (MEDIUM) Large/mega-sized (LARGE)	51.6 37.1 8.8 2.5	Size is measured by means of the number of employees and according to the definition of SMEs provided by the European Union recommendation 2003/361
Prevalence of commercial versus social goals in case of conflict (CONLICT)	Commercial goals (CONFLICT = 0) Social goals (CONFLICT = 1)	31.8 68.2	The questionnaire asked the respondents to indicate which one of both dimensions had prevailed in case of conflict between the commercial activity and the social mission

## Results

### Reliability and validity of the ‘collaboration continuum’ scale

A confirmatory factor analysis using EQS 6.2 for Windows evaluated the reliability and validity of the collaboration continuum (CC) scale (Steenkamp and Trip 1991). The process showed that this scale was comprised of four reflective sub-dimensions: (1) *strategic nature of the partnership* (importance to mission, strategic value, magnitude of resources, and scope of activities), (2) *relational development* (level of engagement/commitment, level of interaction/communication, and trust), (3) *complexity and change* (managerial complexity, internal change, and external system change), and (4) *synergistic and transformational value* (opportunities for learning/capability development, and development of social innovations/co-creation of value).

Table 4 shows the reliability and validity indicators. Reliability was assessed through the Cronbach's alpha and the composite reliability coefficient: the coefficients exceeded the recommended value of 0.7. There is statistical significance between each item and its factor, and the values of all the standardized coefficients (factor loadings) are greater than 0.5. In turn, the values of the average variance extracted (AVE) coefficients were greater than 0.5 for all constructs, supporting convergent validity (Hair et al. 2009). Since the items of the four sub-dimensions show convergent validity, for each subscale we added their individual item scores to obtain a mean measure for the strategic nature of the partnership, relational development, complexity and change, and synergistic and transformational value. Moreover, as the whole scale presents convergent validity we added these four measures to have a global index of the position of the partnership.

**Table 4:** Reliability and validity of the ‘collaboration continuum’ scale

Dimensions	Items	Factor loadings	Cronbach's alpha	Composite reliability coefficient	AVE
Strategic nature of the partnership (STNATURE)	STN1 STN2 STN3 STN4	0.821*** 0.810*** 0.827*** 0.604***	0.845	0.853	0.595
Relational development (RELDEV)	RD1 RD2 RD3	0.900*** 0.918*** 0.677***	0.871	0.875	0.704
Complexity and change (CHANGE)	CH1 CH2 CH3	0.598*** 0.685*** 0.843***	0.758	0.756	0.512
Synergistic and transformational value (SI)	SI1 SI2	0.930*** 0.631***	0.822	0.768	0.632
Goodness-of-fit measures S-Bχ2 = 95.5136 (*p* = 0.000); S-Bχ2/degrees of freedom = 1.99; BBNNFI = 0.917; CFI = 0.939; RMSEA = 0.08					

***p < .01

### Model results

We estimated five linear regression models using IBM SPSS Statistics 24 software. We used this technique instead of other alternatives such as structural equation modelling because (1) in our research there were not dependent variables that could be predictor variables of other dependent variables, and (2) we were interested in simultaneously analyzing the effect of a wide set of control variables. In each regression, the SE’s accountability to the stakeholder analyzed was the dependent variable. Results are depicted in Table 5 (standardized coefficients and *t* values).

**Table 5:** Effect of the SE-nonprofit partnership on the SE’s accountability to stakeholders

Variables	Beneficiaries	Customers	Employees	Donors	Publicad
Constant	---(2.432)**	---(1.891)*	---(2.243)**	---(3.603)***	---(1.262)
Collaboration continuum (CC)	0.364(3.869)***	0.246(2.459)**	0.150(1.568)	0.072(0.720)	0.152(1.738)*
CLUSTER2	0.058(0.472)	0.174(1.339)	0.010(0.083)	0.222(1.702)*	− 0.044(− 0.388)
CLUSTER3	0.037(0.333)	0.206(1.724)*	0.030(0.264)	− 0.050(− 0.418)	− 0.090(− 0.861)
CLUSTER4	0.020(.180)	0.225(1.938)*	0.018(0.165)	− 0.041(− 0.351)	− 0.151(− 1.495)
Type of business form (TYPE)	− 0.279(− 1.969)**	− 0.226(− 1.497)	− 0.125(− 0.869)	− 0.137(− 0.904)	0.223(1.693)*
Legal form (LEGFORM)	− 0.163(− 1.887)*	− 0.159(− 1.729)*	0.146(1.661)*	− 0.080(− 0.868)	− 0.011(− 0.142)
Promotor: Natural person (PR_NP)	− 0.055(− 0.373)	0.161(1.031)	0.048(0.324)	− 0.043(− 0.277)	− 0.040(− 0.290)
Promotor: Association (PR_ASSOC)	0.134(1.319)	0.096(0.891)	− 0.096(− 0.930)	− 0.165(− 1.526)	0.055(0.585)
Promotor: Foundation (PR_FOUND)	0.122(1.096)	0.095(0.800)	0.033(− 0.290)	− 0.010(− 0.081)	− 0.045(− 0.436)
Promotor: Public Administration (PR_PA)	0.032(0.412)	− 0.034(− 0.403)	0.045(0.561)	− 0.005(− 0.059)	0.099(1.360)
Promotor: Commercial/ Trading business (PR_COMM)	0.015(0.171)	0.216(2.239)**	0.118(1.282)	− 0.061(− 0.625)	0.023(0.270)
Promotor: Social economy enterprise (PR_SE)	0.101(1.082)	0.103(1.036)	− 0.028(− 0.298)	0.178(1.785)*	0.007(0.081)
Promotor: Religious entity (PR_REL)	0.118(1.314)	0.108(1.134)	0.074(0.807)	0.127(1.328)	0.033(0.391)
Culture/ recreation (ACT_CUL)	0.072(0.902)	0.012(0.142)	− 0.037(− 0.455)	− 0.059(− 0.685)	− 0.017(− 0.233)
Education (ACT_EDU)	0.117(1.420)	0.049(0.555)	0.251(3.004)***	0.039(0.447)	− 0.032(− 0.413)
Research (ACT_RES)	− 0.289(− 3.236)***	− 0.150(− 1.581)	0.043(0.471)	− 0.038(− 0.394)	− 0.076(− 0.916)
Environment (ACT_ENV)	− 0.041(− 0.488)	− 0.008(− 0.089)	− 0.052(− 0.612)	− 0.061(− 0.687)	0.073(0.939)
International development cooperation (ACT_COOP)	0.035(0.409)	− 0.057(− 0.627)	0.195(2.234)**	0.134(1.462)	− 0.085(− 1.070)
Religion (ACT_REL)	0.020(0.243)	0.072(0.840)	0.054(0.656)	0.066(0.765)	0.022(0.297)
Local development and housing (ACT_HOUS)	− 0.046(− 0.578)	− 0.021(− 0.244)	0.037(0.455)	0.081(0.951)	− 0.084(− 1.129)
Health (ACT_HEALH)	0.081(0.972)	− 0.069(− 0.774)	− 0.032(− 0.376)	0.239(2.688)***	0.117(1.508)
Business, professional associations, unions (ACT_ASSO)	0.064(0.728)	0.029(0.306)	− 0.141(− 1.576)	− 0.034(− 0.360)	0.215(2.627)***
Law, advocacy and politics (ACT_ADV)	0.038(0.475)	− 0.066(− 0.774)	− 0.058(− 0.705)	− 0.009(− 0.108)	− **0.127(**− 1.706)*
Micro size (MICRO)	− 0.036(− 0.140)	0.001(0.004)	0.720(2.789)***	− 0.159(− 0.585)	− 0.113(− 0.481)
Small size (SMALL)	0.017(0.069)	0.029(0.108)	0.700(2.749)***	− 0.117(− 0.437)	0.259(1.113)
Medium size (MEDIUM)	0.182(1.126)	0.106(0.615)	0.485(2.938)***	− 0.119(− 0.687)	0.247(1.636)*
Prevalence of commercial versus social goals in case of conflict (CONFLICT)	0.010(0.124)	0.092(1.050)	0.062(0.746)	− 0.207(− 2.363)**	0.052(0.681)
	R^2^ = 0.362 Adjusted R^2^ = 0.218 Sig. = 0.000	R^2^ = 0.268 Adjusted R^2^ = 0.105 Sig. = 0.037	R^2^ = 0.332 Adjusted R^2^ = 0.183 Sig. = 0.002	R^2^ = 0.276 Adjusted R^2^ = 0.111 Sig. = 0.031	R^2^ = 0.447 Adjusted R^2^ = 0.322 Sig. = 0.000

*p < 0.10; **p < 0.05; ***p < 0.01

Results support the two hypotheses. The greater the value of CC, the greater the extent to which the SE declares that it is accountable to beneficiaries (BENEFICIARIES: β = 0.364, *p* < 0.01), as H1 expected. Furthermore, the coefficient associated with CC is also positive and significant regarding customers (CUSTOMERS: β = 0.246, *p* < 0.05) and public administrations (PUBLICAD: β = 0.152, *p* < 0.10). In the case of employees (EMPLOYEES) and donors/funders (DONORS), their coefficients are positive although they are not statistically significant. Overall, these findings show that as the partnership evolves along the continuum toward the transformational stage, accountability to beneficiaries improves significantly without hindering accountabilities to funders/donors, employees, clients, and public administrations (H2).

The different configurations of resources do not affect in a significant fashion accountability to beneficiaries, but they impact on accountability to customers (CUSTOMERS) and donors (DONORS). Compared to those collaborations in which SEs and NPOs provides the five categories of resources to a lesser extent (CLUSTER1), CLUSTER3 and CLUSTER4 reinforce accountability to customers, and CLUSTER2 strengthens accountability to donors.

An additional noteworthy result refers to the effect linked to the type of SE (WISE versus more entrepreneurial SEs created after the crisis of 2008). Accountability to beneficiaries is significantly improved when the SE belongs to this second category of SEs (β = − 0.279, *p* < 0.05). On the contrary, WISEs are SEs that show a greater accountability to public administrations.

The individual analysis of each of the five regression models shows other insights:

The accountability to beneficiaries is also greater in SEs whose legal form corresponds to commercial businesses (LEGFORM), and in those cases in which the social area of activity does not include research (ACT_RES). If we focus on accountability to customers, as occurs with beneficiaries, commercial businesses (LEGFORM) also present a positive impact, as well as those SEs which promoters are commercial businesses (PR_COMM).

Unlike beneficiaries and customers, the accountability to employees is greater in SEs that adopt the legal form of a cooperative or a worker-owned company (LEGFORM). SEs focused on education (ACT_EDU), international development cooperation (ACT_COOP), or those characterized by being micro, small or medium-sized organizations (MICRO, SMALL, MEDIUM) also attribute greater accountability to employees.

SEs whose promoters have been organizations from the social economy (PR_SE) or SEs focused on health-related activities (ACT_HEALTH) assign more relevance to donors/investors. This type of accountability is also perceived in those companies in which the commercial activity has prevailed over the social mission in case of conflict (CONFLICT).

Finally, SEs which activity is related to business and professional associations/unions (ACT_ASSO) or those that are medium in size recognize that they are accountable towards public administrations as a relevant stakeholder to a greater extent. Advocacy-related activity presents a significant negative effect (ACT_ADV).

## Conclusions and implications

This study has analyzed the impact of partnerships between SEs and NPOs on SEs’ accountability. The results show that SEs engaged in partnerships characterized by an integrative or (even more) transformational nature, tend to encourage accountability to (principally) beneficiaries of the social mission, but without undermining their accountability to other types of relevant stakeholders. These insights lead to some remarkable conclusions.

First, the study makes evident that not all collaborations between SEs and NPOs affect the individual partners’ organizational models in the same way. The characteristics of the partnership with regard to a range of factors that define its position along a collaboration continuum change the degree to which the SE recognizes that it is accountable to its different groups of stakeholders. As the partnership moves toward the end of the continuum, co-creation activities associated with the transformational stage enhance the relevance of beneficiaries, but also customers, and even public administrations, broadening the scope of the stakeholders to which the SE is accountable, beyond those investor and donor groups that control access to resources. At the same time, accountability to donor/investors and to employees is not affected.

Second, customers and beneficiaries gain importance as the partnership moves along the continuum, but the effect seems to be stronger in the case of beneficiaries. This insight is noteworthy because, although in business literature the importance of customers has been extensively remarked, this wide recognition does not occur in the case of beneficiaries. The weak standing of beneficiaries in terms of (even) non-profit accountability has been highlighted by the impact measurement literature (Wellens and Jegers 2016). Our results support the idea that the more transformational partnerships involving SEs and NPOs become, the more accountable SEs feel towards beneficiaries, thus diluting any eventual dichotomy or trade-off between the targets of their commercial efforts and those of their social mission.

Therefore, while literature usually associates the concept of hybrids with irreconcilable trade-offs between social and commercial goals, our results show that it is possible that under certain conditions one goal does not necessary compromise the other, in line with Muñoz and Kimmitt (2019). The consolidation of relationships with NPO in the terms described could promote the generation of additional financial resources and other types of support, which in turn could favor better insights into how to optimize operations. Supporting this expectation, our results suggest that SEs that are engaged in collaborations with NPOs in which both partners provide a wider range of resources are also organizations that reinforce accountability to external providers of financial resources and revenues.

Third, recent literature on business-nonprofit partnerships has focused their attention on investigating the organizational capabilities and routines that partners need to manage these collaborations, adopting the perspective of the nonprofit. For instance, Liu et al. (2018) analyze the effect of five basic alliance management routines on alliance performance, as well as the mediating effect of three relational mechanisms. In a complementary way, Al-Tabbaa et al. (2019) identifies 27 first-order capabilities, and incorporates a time dimension to group them into 15 themes. Although these two studies represents examples of opposite positions regarding whether such capabilities should be perceived as being adaptive rather than universalistic (the former defends that cross-sector alliance literature can learn much from strategic (firm-firm) management; the latter emphasizes that capabilities depend of the type of partnerships), overall, both show that relational skills are necessary to foster the partnership performance, as well as the importance of an appropriate management of stakeholder groups. Our research, considering the view of the social enterprise instead of the NPOs, supports these insights.

Finally, the current study also reveals that the type of business and legal forms influences the extent to which the SE is accountable to different kinds of stakeholders, an issue that has been little explored in previous literature (Saebi et al. 2019). Supporting studies that note that WISEs are SEs in which commercial and social activities are usually less integrated, a fact that leads to trade-offs between them (Battilana and Lee 2014), we have found that accountability to beneficiaries significantly decreases when the SE is a WISE compared with other forms of SEs. For its part, SEs that adopt commercial business legal forms seems to present a greater accountability to beneficiaries/customers, maybe because the competition they face encourages their customer-centric orientation, whereas SEs that adopt legal forms of the social economy show a greater accountability to employees.

Some practical implications can be also derived from the results obtained.

If the goal of a SE is to gain legitimacy towards stakeholders beyond investors/donors and employees, it should attempt to foster a type of partnership with NPOs that is (1) deep-rooted in the strategic core activities of both organizations, (2) characterized by a high degree of engagement/commitment, trust, and interaction/communication, (3) managed in a professional and appropriate manner to implement the required organizational changes, and (4) aimed at promoting social innovation and co-creation. And to foster such a kind of partnership, the following suggestions for practitioners can be highlighted:

First, it would be relevant to face those frequent barriers that prevent collaborations become strategic and linked to the core activities of both organizations, especially regarding the lack of staff capacity to manage cross-sector collaborations, or the lack of a robust process of design and implementation of the partnership. The detailed and practical guideline proposed by The Partnering Initiative (2020), in collaboration with United Nations, can be useful to help SEs and NPOs build high-value partnerships.

Second, trust and commitment can be encouraged by means of activities that develop a mutual understanding (e.g., encouraging temporary personnel mobility among organizations, personal contacts, reporting procedures). In addition, to improve information flows, partners should know the particular requirements derived from the environment of each organization, so that each organization understands the operations and decision-making of the partner.

Third, transformational partnerships are also characterized by a process of internal change. To manage this change, top management commitment of both SEs and nonprofits as well as a strategy of change management will be critical.

Fourth, co-creation processes could be enhanced with activities such as improving the participation of the partner in the different activities of the organization, showing a positive attitude towards the influence of the other party, incorporating the opinions and suggestions provided by the partner into the procedures and routines of the organization through a dynamic learning process, and fostering the existence of an effective involvement of the staff of both organizations.

The results of the research can also provide implications for policy makers. The need to move from an industrial to a knowledge-based economy in which hybridity and interconnectivity are the ‘new normal’ not only demands programs aimed at developing the digital transformation, but also training in those ‘soft’ skills needed to implement cross-sector partnerships. For example, educational programs aimed at encouraging entrepreneurship capabilities should include in their syllabus competences required to foster the experience and knowledge about the three sectors (for-profit, nonprofit, and public sectors), networking skills, conflict resolution capabilities, and experience in the development of informal relationships and change management.

Furthermore, the role that NPOs can play in encouraging accountability to beneficiaries (and therefore social innovations) suggests the interest of developing policies aimed at reinforcing the creation and maintenance of these organizations. In this regard, policymakers should promote policies that attempt to alleviate those characteristics of nonprofits that generate barriers to citizens’ involvement, for example policies that enhance the development of good-governance practices and reporting mechanisms to foster transparency in these organizations.

## Limitations and further research

The research is not free of limitations. The first one refers to the sample. We have distinguished between WISEs and other SEs which tend to adopt commercial legal forms and better integrate their social mission with their entrepreneurial activity. Nevertheless, our data do not allow us to differentiate the whole range of possible hybrid models. In this regard, Santos et al. (2015) have identified four basic groups of models: (1) market hybrids (customers are also the beneficiaries, and social value spillovers are created by the same market-based activities); (2) blending hybrids (customers and beneficiaries are the same target, but creating social value requires different activities); (3) bridging hybrids (in which the organization attempts to create social value for beneficiaries who are different from the customers, through the same activities carried out for obtaining financial outcomes); and (4) coupling hybrids (which use differentiated models to serve beneficiaries and customers).

Second, the research has focused only on the SE self-perceived accountability, but neither the nonprofits’ nor the stakeholders’ viewpoints have been directly assessed.

Addressing these gaps can serve as possible ways to advance in this research. Moreover, two additional possibilities can be noted.

The former is related to value co-creation in SEs. In the current research we refer to value co-creation in general terms, meaning activities such as the participation of the stakeholders in the different stages of the decision process, the fact that there is reciprocity between the partners, the existence of a dynamic learning process, and the existence of an effective engagement of the SE with the NPO to foster a long-term relationship. It would be also noteworthy to focus this broad perspective on exploring the effect of partnerships between SEs and NPOs on the so-called ‘brand value co-creation’ (Ramaswamy and Ozcan 2016). As Ramaswamy and Ozcan (2016, p. 97) highlight, “brands are now increasingly seen in light of collaborative, value creation activities of a firm and all of its stakeholders, and brand value as a collective measure of all stakeholders’ perceived values.” SEs could obtain important benefits from co-creating brand value with NPOs and beneficiaries, and further research can be devoted to investigate the required activities involved in that process.

The second potential line for future research could address the effect of the fit between the internal conditions of the partnerships (e.g., social context factors such as trust, commitment, level of communication, or other internal conditions such as, for example, formalization) and different collaboration forms (cause-related marketing campaigns, sponsorships, corporate volunteering programs, joint ventures, etc.) on partnership performance and results, in line with what other previous works have carried out (Murray and Kotabe 2005).

## Appendix

See Tables

**Table:** Dependent variables (measured in a seven-point Likert scale)

Accountability to:		Mean(Standard Deviation)
Employees (EMPLOYEES)		5.50(1.365)
EMPL1	To what extent employees contribute to define what the success of the social enterprise is (objectives, priorities, performance, …)	5.82(1.470)
EMPL2	To what extent the social enterprise is accountable to employees	5.19(1.688)
Investors/donors (DONORS)		4.41(2.015)
DON1	To what extent investors/donors contribute to define what the success of the social enterprise is (objectives, priorities, performance, …)	4.01(2.119)
DON2	To what extent the social enterprise is accountable to investors/donors	4.82(2.264)
Public administrations (PUBLICAD)		4.03(1.665)
PA1	To what extent public administrations contribute to define what the success of the social enterprise is (objectives, priorities, performance)	3.55(1.932)
PA2	To what extent the social enterprise is accountable to public administrations	4.50(2.065)
Beneficiaries of the social mission (BENEFICIARIES)		4.59(1.559)
BEN1	To what extent beneficiaries contribute to define what the success of the social enterprise is (objectives, priorities, performance, …)	4.99(1.857)
BEN2	To what extent the social enterprise is accountable to beneficiaries	4.20(1.897)
Customers of the commercial activity (CUSTOMERS)		4.48(1.523)
CUST1	To what extent customers contribute to define what the success of the social enterprise is (objectives, priorities, performance, …)	4.92(1.862)
CUST2	To what extent the social enterprise is accountable to customers	4.05(1.928)

**Table:** Independent variable (measured in a seven-point Likert scale)

Collaboration continuum (CC) Please, describe the partnership in terms of the following factors:		Mean(Standard deviation)
*Strategic nature of the partnership (STNATURE)*		4.60(1.443)
STN1	Importance to mission (from peripheral to central)	4.96(1.737)
STN2	Strategic value (from minor to major)	4.96(1.664)
STN3	Magnitude of resources (form small to big)	4.27(1.634)
STN4	Scope of activities (from narrow to broad)	4.21(1.938)
*Relational development (RELDEV)*		5.42(1.209)
RD1	Level of engagement/commitment (from low to high)	5.24(1.456)
RD2	Level of trust (from modest to deep)	5.80(1.185)
RD3	Level of interaction/communication (from infrequent to intensive)	5.21(1.414)
*Complexity and change (CHANGE)*		3.95(1.395)
CH1	Managerial complexity (from simple to complex)	3.97(1.631)
CH2	Internal change (from minimal to great)	3.44(1.763)
CH3	External system change (from rare to common)	4.43(1.703)
*Synergistic and transformational value (SI)*		4.99(1.640)
SI1	Synergistic value: opportunities for learning and capability development (the extent to which the partnership has generated this type of value)	5.04(1.691)
SI2	Development of social innovations / co-creation of value (the extent to which the partnership has generated this type of value)	4.94(1.865)

Type of resources (from “1 = the organization has not contributed with this type of resource at all” to “7 = this type of resource represents a critical contribution to the agreement”)	Mean (Standard deviation)	
	Social enterprises	Nonprofits
Human resources: paid employees and/or volunteers	5.03(1.854)	4.12(2.014)
Financial resources: cash	3.42(1.947)	2.92(2.107)
In-kind resources: infrastructure (furniture, accommodation, technical equipment, etc.), office supplies, services, etc	3.58(1.749)	3.52(1.907)
Relational-based resources: networks of contacts, information and knowledge about the environment, brand image or reputation	4.83(1.435)	4.80(1.542)
Internal capabilities: technical training, experience in management, experience in project development, promotion of projects	4.69(1.494)	4.27(1.618)
